# Data set for characterization of TNF-α–inducible glycosphingolipids in vascular endothelial cells

**DOI:** 10.1016/j.dib.2018.09.059

**Published:** 2018-09-26

**Authors:** Tetsuya Okuda

**Affiliations:** Bio-Design Research Group, Bioproduction Research Institute, National Institute of Advanced Industrial Science and Technology (AIST), Central 6, 1-1-1 Higashi, Tsukuba, Ibaraki 305-8566, Japan

## Abstract

The data presented here pertain to a research article entitled “Structural characterization and dynamics of globotetraosylceramide in vascular endothelial cells under TNF-α stimulation” (Okuda et al., 2010). The present article provides additional structural and gene expression data for the characterization of a TNF-α–inducible glycosphingolipid, globotetraosylceramide (Gb4), in vascular endothelial cells. (i) Structural details of Gb4 in lipid raft–enriched cell membranes were determined by MALDI-TOF MS. These analyses identified Gb4 with very-long-chain fatty acids as the major molecular species in this fraction, and the expression levels of whole molecular species of Gb4 with different fatty acid structures in the membrane are uniformly upregulated by TNF-α stimulation. (ii) The expression levels of genes encoding enzymes for synthesis of the ceramide portion of Gb4 were analyzed by real-time PCR. These assays revealed that TNF-α stimulation promotes transcription of the *Elovl1* and *Cers5* genes, which are involving in the synthesis of Gb4 with very-long-chain fatty acids. Collectively, these results indicate that TNF-α regulates glycosphingolipid synthesis and lipid raft formation in vascular endothelial cells via transcriptional up-regulation of related genes. These data thus provide new insights useful for understanding the molecular basis of inflammation-associated pathology in vascular endothelia.

**Specifications table**

**Table:** 

Subject area	*Cell biology*
More specific subject area	*Vascular endothelial cells, inflammation, glycosphingolipids, lipid rafts, fatty acid elongase, ceramide synthase*
Type of data	*Images and tables*
How data was acquired	*MALDI-TOF MS and real-time PCR*
Data format	*Raw and analyzed data*
Experimental factors	*Vascular endothelial cells stimulated by TNF-*α.
Experimental features	*Lipid raft–enriched cell membranes were prepared by sucrose density gradient centrifugation in the presence of Triton X-100; purified membrane-associated glycosphingolipids were analyzed by MALDI-TOF MS using* α*-CHCA as the matrix.*
	*cDNA was prepared from total cellular RNA, and the expression level of target genes was analyzed by real-time PCR using fluorescent-labeled hydrolysis probes.*
Data source location	*Bioproduction Research Institute, National Institute of Advanced Industrial Science and Technology (AIST), Central 6, Tsukuba*
Data accessibility	*The data are available with this article.*
Related research article	*T. Okuda, et al. Structural characterization and dynamics of globotetraosylceramide in vascular endothelial cells under TNF-α stimulation. Glycoconj. J. 27 (2010)287–296.*[1]

**Value of the data**

• The data provide new insights into the molecular dynamics of glycosphingolipids in lipid rafts under inflammatory conditions, which can be of value to researchers in related fields.
• Transcriptional up-regulation of *Elovl1* and *Cers5* in response to TNF-α stimulation is a novel finding.
• These data can be compared to other scientific data addressing the effects of TNF-α on various cells and tissues.
• The data and protocols provided here support other researchers investigating vascular inflammation and glycosphingolipids.

## 1 Data

**Fig. 1:**
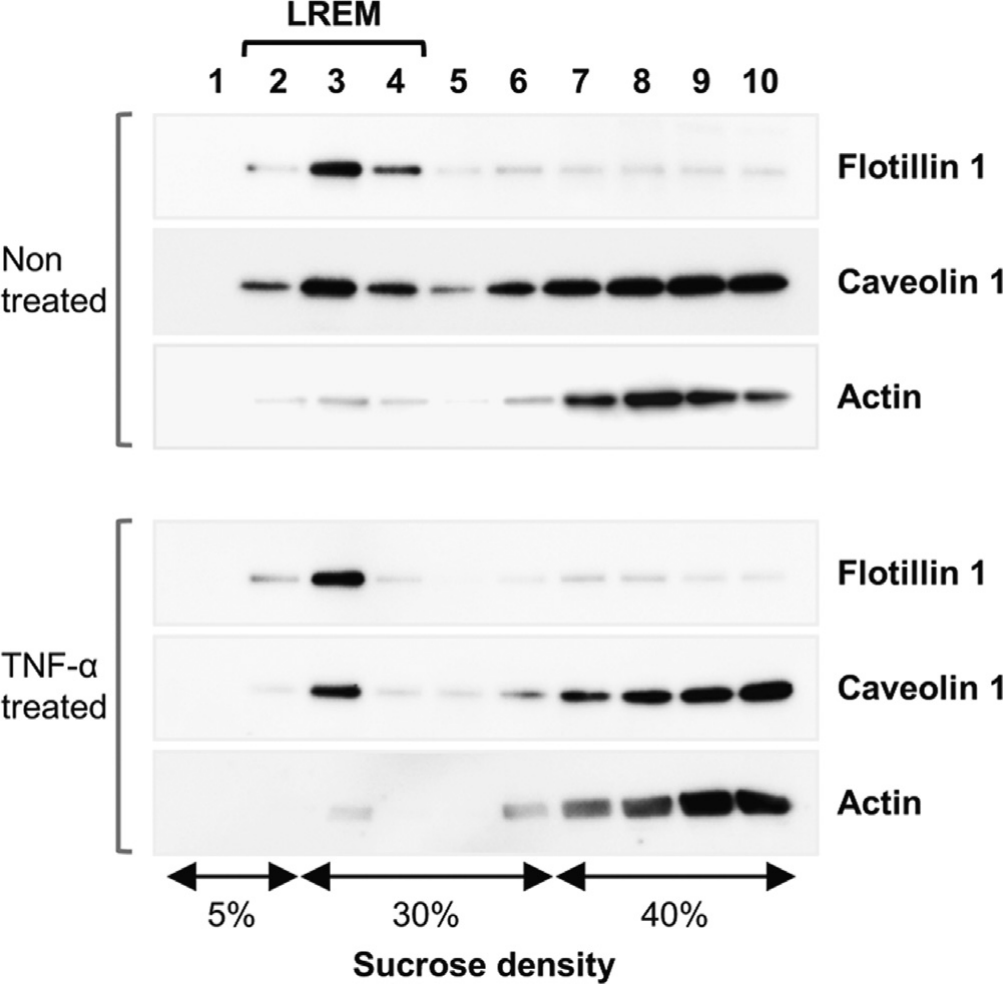
Prepared lipid raft–enriched membrane fraction. Lipid raft markers, flotlline 1 and caveolin 1, were detected by immunoblotting, as described in the Section 2. Actin was used an internal control. LREM, lipid raft–enriched membrane.

**Fig. 2:**
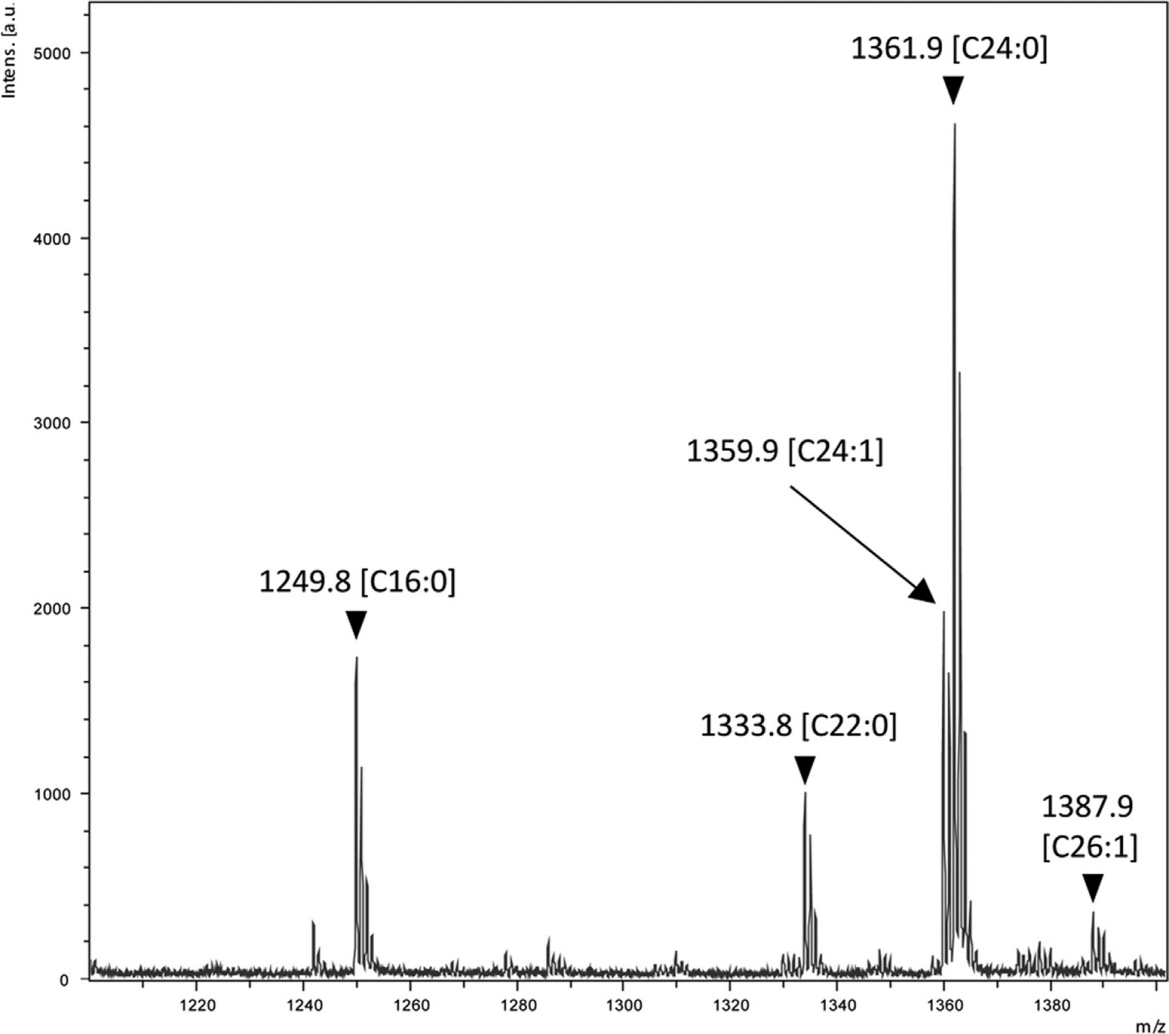
Representative mass spectrum of Gb4 purified from a lipid raft–enriched membrane.

**Fig. 3:**
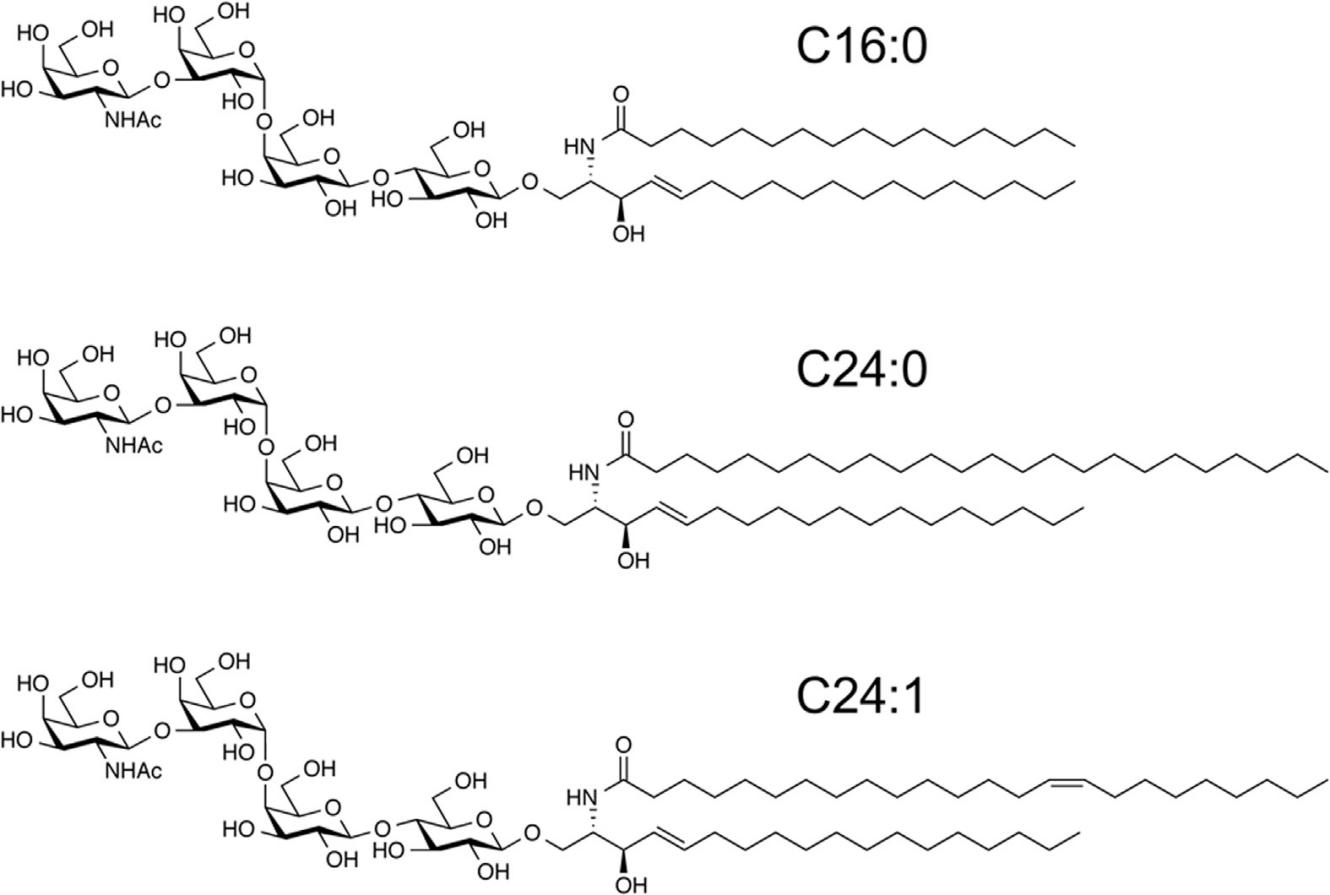
Chemical structures of Gb4 with C16:0 or C24 fatty acids.

For MALDI-TOF MS analyses, lipid raft–enriched membranes (LREMs) of non-treated vascular endothelial cells (ECs) were prepared by sucrose density gradient centrifugation using 1% triton X-100, and fractions enriched in the lipid raft marker flotlline 1 were isolated as the LREM (Fig. 1). The LREM also partially included the caveolae marker caveolin 1. Glycosphingolipids were purified from the LREM fraction and the molecular composition of globotetraosylceramide (Gb4), a TNF-α–inducible glycosphingolipid in EC [1], was determined by MALDI-TOF MS. In positive-ion mass spectra, molecular ions corresponding to Gb4 were detected: [M+Na]^+^ (*m/z* 1249.8–1387.9). A representative mass spectrum of Gb4 in the LREM is shown in Fig. 2, and the results are summarized in Table 1. Major peaks were detected at *m/z* 1249.8, 1359.9, and 1361.9, corresponding to Gb4 with 4-sphingosine and C16:0 fatty acid, C24:1 fatty acid, and C24:0 fatty acid, respectively. These chemical structures are shown in Fig. 3. Minor peaks were detected at *m/z* 1333.8 and 1387.9, corresponding to Gb4 with 4-sphingosine and C22:0 fatty acid and C26:1 fatty acid, respectively. The MS data were statistically compared with data pertaining to TNF-α–treated ECs [1]. The intensity of these signals increased uniformly in TNF-α–treated ECs, and the total signal intensity increased significantly, by over 2-fold, compared to that of non-treated ECs. In contrast to the whole cell membrane [1], the ratio of these molecular species of Gb4 was unchanged, and the ratio in the LREM of ECs was close to that in the whole cell membrane of TNF-α–treated ECs [1].

**Table 1:** Mass spectra of Gb4 detected in LREMs.

MS	Ceramide	Relative rate (%)		Signal intensity (×10^−3^)	
[M+Na]^+^		Non	TNF	Non	TNF
1249.8	C16:0	18.25 ± 4.68	13.31 ± 5.27	1.62 ± 0.13	2.72 ± 1.62
1333.8	C22:0	10.46 ± 1.70	11.39 ± 0.89	0.98 ± 0.34	2.32 ± 0.78
1359.9	C24:1	22.24 ± 3.31	26.51 ± 0.73	2.00 ± 0.17	5.33 ± 1.59
1361.9	C24:0	45.36 ± 5.79	44.37 ± 5.58	4.23 ± 1.35	8.86 ± 2.58
1387.9	C26:1	3.69 ± 0.40	3.42 ± 0.74	0.34 ± 0.10	0.68 ± 0.21
			Total	9.17 ± 1.90	20.1 ± 5.88*

Means ± S.D.; *n* = 3.

* *P* < 0.05, non-treated cells vs TNF-α–treated cells. Non, non-treated cells; TNF, TNF-α–treated cells.

For real-time PCR analyses, we examined six candidate genes (*Elovl1, Elovl3, Elovl6, Cers2, Cers5,* and *Cers6*) involved in the synthesis of Gb4 with C16 or C24 fatty acids. *Elovl1* and *Elovl3*, which encode fatty acid elongase 1 and 3, respectively, mediate elongation of C18:0 and C20:1 acyl-CoAs to very-long-chain fatty acids, including C24:0 and C24:1 [2]. *ELovl6* encodes fatty acid elongase 6, which mediates elongation of C16:0 acyl-CoA to C18:0 and C20:1 [2]. The *Cers2* gene encodes ceramide synthase 2, which catalyzes the incorporation of C24:0 and C24:1 acyl-CoAs into sphingosine [3]. *Cers5* and *Cers6* encode ceramide synthase 5 and 6, respectively, which catalyze the incorporation of C16:0 acyl-CoA into sphingosine [3]. Ceramide synthase 5 can dimerize with ceramide synthase 2 to enhance the activity of ceramide synthase 2 [4]. The results of real-time PCR analyses are summarized in Table 2. *Elovl1* transcripts were clearly detected in non-treated ECs, whereas *Elovl3* transcripts were barely detectable, and *Elovl1* expression was significantly up-regulated by TNF-α stimulation. TNF-α stimulation tended to increase the expression of *Cers2*, and significant up-regulation of *Cers5* expression was observed following TNF-α stimulation. These results correlate with increased expression of Gb4 with C24 fatty acids in TNF-α–treated ECs. No significant changes were observed in the expression levels of other target genes, indicating that up-regulated expression of Gb4 with C24 fatty acids is regulated primarily by *Elovl1* and *CerS5*.

**Table 2:** Real-time PCR analysis of target gene expression.

	Relative expression		TNF/Non	*P*-value
	Non	TNF		
*Elovl1*	3.41 ± 0.71	58.64 ± 17.39	17.20	0.008**
*Elovl3*	n.d.	n.d.		
*Elovl6*	2.66 ± 1.41	2.96 ± 2.07	1.11	0.820
*Cers2*	0.17 ± 0.04	2.46 ± 1.55	14.75	0.059
*Cers5*	0.64 ± 0.08	15.97 ± 5.48	24.98	0.011*
*Cers6*	2.87 ± 0.89	5.04 ± 1.83	1.75	0.095

The relative expression of target genes is shown as the ratio of expression relative to that of the internal control gene, *St3gal5*. As the level of *St3gal5* expression in non-treated and TNF-α–treated ECs was more stable than that of general reference genes (*Gapdh* and *Actb*), it was used as the internal control in this experiment. Mean ± S.D.; *n* = 4 from two independent experiments.

* *P*< 0.05,

** *P* < 0.01, non-treated vs TNF-α–treated. Non, non-treated cells; TNF, TNF-α–treated cells; n.d., not detected.

## 2 Experimental design, materials, and methods

### 2.1 Cell culture

ECs (HUVEC; KURABO, Osaka, Japan) were maintained in HuMedia-EG2 (KURABO) culture medium, as reported previously [5]. The medium was replaced with fresh HuMedia-EG2 containing 20 ng/ml of TNF-α, and the cells were incubated for 12 or 24 h prior to MALDI-TOF MS or real-time PCR analysis, respectively.

### 2.2 Preparation of LREMs

The detergent-insoluble membrane fraction was isolated from ECs stimulated with TNF-α using sucrose density gradient centrifugation in the presence of Triton X-100 [1]. Protein markers for caveolae/lipid raft or actin protein in each fraction were detected by immunoblotting using mouse IgG1 monoclonal antibodies (BD Biosciences, Franklin Lakes, NJ), anti-caveolin 1 (mAb 2297), anti-flotilline 1 (mAb 18), and anti-Actin (mAb C4) as appropriate.

### 2.3 Glycosphingolipid extraction

Glycosphingolipid extraction from LREM was performed as reported previously [1]. In brief, total lipids from the LREM were sequentially extracted using the chloroform/methanol/water 2:1:0 and 1:2:0.8 (*v*/*v*/*v*), respectively, and glycosphingolipids were separated by column chromatography using Iatrobeads 6RS-8060 (Mitsubishi Kagaku Iatron, Tokyo, Japan).

### 2.4 MALDI-TOF MS

Mass spectrometry analysis of glycosphingolipids was carried out using MALDI-TOF MS according to a previously published method [1].

### 2.5 Real-time PCR

Real-time PCR analyses were performed as reported previously [6] with slight modifications which used fluorescent-labelled hydrolysis probes. Amplification and quantification of target gene cDNAs were performed using a Light Cycler® 480 II system with gene-specific primers, single hydrolysis probes (Universal ProbeLibrary Probes; Roche, Basel, Switzerland) and a reaction mixture containing Light Cycler® 480 Probe Master (Roche), according to the manufacturer׳s instructions. The gene-specific primers and single hydrolysis probes used in this study are summarized in Table 3.

**Table 3:** Primers and probes used for real-time PCR analyses.

Gene (number^a^)	Primer sequence	Probe sequence (Probe number^b^)
*Elovl1*	*Forward: 5*′*-ACTTCTCTCTGGCCCTGCT*	*GGATGGAG*
(NM_022821)	*Reverse: 5*′*-TCACCTCTTGGTACAAGTTCACA*	*(#58)*
*Elovl3*	*Forward: 5*′*-AACCTGCAAGGGCCTCTC*	*CTTCTGCC*
(NM_152310)	*Reverse: 5*′*-TAATGCCCCACATCCTCACT*	*(#29)*
*Elovl6*	*Forward: 5*′*-TTTGAACTGAGGAAGCCATTAGT*	*CTGGTCTC*
(NM_024090)	*Reverse: 5*′*-CAGTTCGAAGAGCACCGAAT*	*(#54)*
*Cers2*	*Forward: 5*′*-AGACGGAGTACACGGAGCAG*	*GCTCCAGA*
(NM_022075)	*Reverse: 5*′*-CGTTCCCACCAGAAGTAATCA*	*(#50)*
*Cers5*	*Forward: 5*′*-CACATCCTCTCGGTGTTCC*	*GGCGGCGG*
(NM_147190)	*Reverse: 5*′*-CAGGGTTTGGCAATAAATCG*	*(#70)*
*Cers6*	*Forward: 5*′*-TCATGATTCAGCTGATGCTCTT*	*GGAGGCTG*
(NM_203463)	*Reverse: 5*′*-CACATTTTCTGAAACTTGGCATA*	*(#75)*
*St3gal5*	Forward: 5′-CTGCCTTTGACATCCTTCAGT	CTGGGGCC
(NM_003896)	Reverse: 5′-CGATTGTGGGGACGTTCTTA	(#57)

a Genbank accession number (http://www.ncbi.nlm.nih.gov/).

b Probe number from the Universal Probe Library (Roche).

### 2.6 Statistical analysis

After determination of variance by the *F*-test, statistical significance was determined using the two-tailed Student׳s *t*-test, with statistical significance defined as follows: *P < 0.05, **P < 0.01.
